# The Influence of the Initial Treatment of Oak Wood on Increasing the Durability of Exterior Transparent Coating Systems

**DOI:** 10.3390/polym15153251

**Published:** 2023-07-30

**Authors:** Ondřej Dvořák, Monika Sarvašová Kvietková, Kristýna Šimůnková, Ondřej Machanec, Miloš Pánek, Filip Pastierovič, Chia-Feng Lin, Dennis Jones

**Affiliations:** 1 Department of Wood Processing and Biomaterials, Faculty of Forestry and Wood Sciences, Czech University of Life Sciences, Kamýcká 1176, 165 00 Prague, Czech Republicdennis.jones@ltu.se (D.J.); 2 Department of Engineering Sciences and Mathematics, Wood Science and Engineering, Lulea University of Technology, Forskargatan 1, SE-931 87 Skellefteå, Sweden

**Keywords:** oak wood, pretreatments, transparent coatings, exterior, durability

## Abstract

This study determined the impact of undertaking an initial treatment of oak wood by sealing its surface pores with epoxy resin, focusing on the durability of transparent coating systems when exposed outdoors. Throughout the exposure period, various parameters including color, gloss, surface wettability, and both macroscopic and microscopic surface evaluation were continuously monitored. The study involved two sets of samples: one set underwent the pretreatment, while the other did not. Subsequently, four coating systems were applied to the samples, comprising two solvent-based and two water-based coatings. The experiment was conducted over a period of two years, utilizing natural weathering methods within the premises of the Czech University of Life Sciences in Prague. The pretreatment with epoxy resin exhibited enhanced durability for all paint systems. The analysis showed a significant difference in gloss and color after 12 months of weathering exposure without any significant effect on surface wettability and sealing. However, after 24 months of the weathering exposure, no significant differences between the sealed and unsealed surface were observed. The most significant change in properties was noted for the water-based coatings used in coating systems number 3 and 4, and these coatings were rated as the best.

## 1. Introduction

Wood, as a natural material, can have a significant impact on the durability of external transparent coating systems. Its properties and behavior vary depending on its species, origin, quality, and conditions [1,2]. The use of wood products in exterior applications is increasingly popular. However, the effects of these exterior conditions, e.g., weathering, require additional protection to prolong the service life of wood [3]. From a practical point of view, chemical protection in the form of paints is often the most preferred method [4]. When dealing with wood that has a distinctive pattern, transparent or semi-transparent coatings are often desired to protect the surfaces [5]. Consequently, research on the improvement of these coatings has been on the rise to meet the growing demand [6]. However, compared to pigmented coating, these types of coatings present durability issues under exterior conditions and require more frequent and extensive maintenance [6,7]. More significant difficulties arise when applying coatings to wood species with specific anatomical structures, such as ring-porous wood species, and wood species with a high content of extractives. In this study, English oak (*Quercus robur* L.) was selected based on its specifications [8]. Oak wood is a popular choice due to its durability, high density, strength, and aesthetic appeal [4]. Oak’s natural durability, according to EN 350, falls in durability classes 1–2 (lab tests) and DC 2–4 (soil test). It is durable against beetles but only moderately durable against termites. Oak wood is susceptible to degradation when exposed to environmental factors such as moisture and UV radiation [9]. Oak wood can be chemically treated with preservatives to protect against mold, rot, and insects [1].

**Figure 1:**
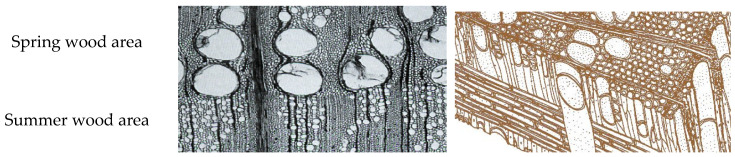
Microscopic images of oak wood. Microscopic sections 40× magnification: transverse section (**left**), 3D structure of oak (**right**) [5].

Several factors can impact the durability of exterior transparent coating systems, including wood species, wood moisture content, type of coating used, surface adhesion, and the susceptibility of the surface to cracking and warping when exposed to moisture and UV radiation [8]. These factors can compromise the adhesion and performance of transparent coating systems [7]. English oak, known for its naturally high resistance to biotic pests according to EN 350 [9], is suitable for outdoor use mainly due to the high occurrence of extractives, which complicates the use of coating systems for protection [10]. Oak wood contains inorganic compounds (oxides and salts) and organic compounds (extractives) [4]. The organic extractives, which can be extracted from wood using various solvents, affect the color and natural resistance of the wood but have a negative effect on any surface treatments applied [1]. These extractives are susceptible to UV radiation, which can lead to surface color change; tannins, for example, can provide short-term resistance but break down over time. Furthermore, these soluble substances have a strong negative effect on surface wetting [11,12]. They can oxidize and delay the curing time of the first coating layer [4,13]. Additionally, tannins react with metals, resulting in the formation of dark spots on the surface upon contact. Evans et al. reported that over 20 different types of hydrolyzable tannins can be isolated from summer oak; these tannins usually contain a polyhydric alcohol, e.g., D-glucose [4,14]. Oak wood tannins also affect wood acidity, with heartwood pH values typically ranging around 3, notably lower than most other woods, which typically exhibit pH values ranging from 4 to 5 [15]. In some cases, the pH value can negatively affect the quality of the coating [4]. Furthermore, the porous structure of pedunculated oak (shown in Figure 1) complicates its treatment [5].

The cross-section of oak wood in Figure 1 shows large vessels in the springwood and smaller vessels in the summerwood [5]. The diameter of spring vessels in oak ranges from 150 to 350 μm, occasionally reaching sizes up to 1 mm, while summer vessels typically have diameters ranging from 30 to 140 μm. Additionally, the length of oak wood fibers is generally up to 1.74 mm [5]. Achieving adhesion of paint materials to oak wood can be problematic due to the presence of these large pores [13], which allow water-soluble extractives, such as tannins, to migrate to the surface to a significant extent. Subsequently, these extractives can react with the coating system, resulting in color changes [16]. Tondi et al. [17] noted that tannins exhibited similar UV-radiation vulnerability to lignins. Although tannins can temporarily protect wood by absorbing UV light, they eventually degrade. Oak wood also contains a high content of tyloses, which can clog vessels and reduce permeability to liquids [18]. However, it was suggested that tyloses have no significant effect on the diffusion of extractives, or at most, have only a minor impact [13].

**Figure 2:**
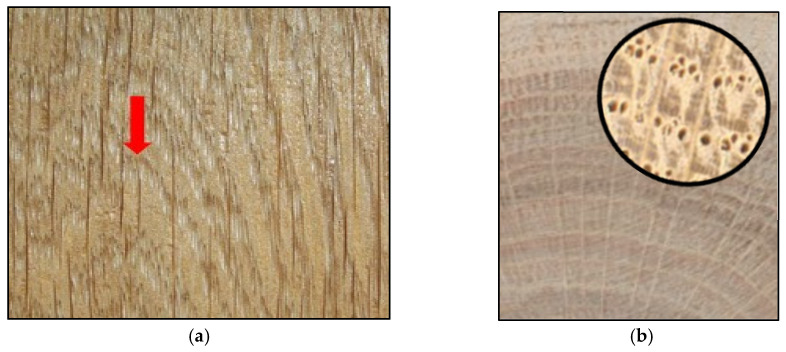
Degradation of oak wood. Degradation of spring wood in oak: (**a**) tangential section showing the faster degradation of springwood in oak, indicated by the red arrow pointed at the large open pores; (**b**) cross-section showing the large pores of oak [7].

The coating system protects the wood against the penetration of moisture, thereby reducing the migration and subsequent leaching of extractive substances and potential cracking risk. Cracks are created due to uneven tension between the inner and outer layers, which allows deeper water penetration and provides an entryway for biotic pests [19,20,21]. Furthermore, the formation of cracks breaks the continuous layer of the paint film, as depicted in Figure 2. The brown lines in Figure 2a represent wider rays, but it is possible to see white areas where the coating layer is broken (indicated by the red arrow in Figure 2). This can lead to peeling of the coating and insufficient protection of the wood [22].

Tangential surfaces tend to be more susceptible to surface layer damage than radial surfaces due to greater shape changes [23]. While transparent coating systems can increase the durability of wood, their effectiveness can vary depending on the surface treatment of the wood. Oak wood, in particular, is known for having a rougher surface compared to other woods, e.g., pine or beech, which can affect the uniformity of coating system application, wettability, and adhesion [2,5,23,24]. Although the exact impact of roughness on coating degradation is not always clear, smoother surfaces generally tend to exhibit better color stability [25,26]. Therefore, appropriate surface preparation, e.g., using pore fillers, can improve coating system quality and level surface acidity and limit the negative effects of pore size [13].

Pore fillers are used to fill large pores in ring-porous woods near the wood surface, creating a continuous and uniform surface for subsequent coating systems. By reducing the migration of extractives to the surface, pore fillers can enhance the effectiveness of the coating [27]. Fillers and primers consist of filler particles and a binder, with the binder commonly based on resins or oils. When using resin-based fillers, it is necessary to choose a binder with an appropriate molecular size to ensure effective penetration into the pores [28]. Compared to oil-based fillers, resin-based fillers have the advantage of faster drying times, typically hardening within 1 h, while oil-based fillers often require up to 24 h to dry [29].

One of the primary challenges associated with transparent coatings is their transmittance of visible and UV radiation, which can lead to the photodegradation of lignin and the generation of free radicals [25]. UV radiation can cause cleavage of covalent bonds in lignin, resulting in the formation of reactive radicals [17,18,19]. These reactive radicals may penetrate deeper into the wood, initiating chain reactions that can extend up to a depth of 2500 µm [30]. While the use of pigments can prevent photodegradation, it is often undesirable from an aesthetic perspective as they cover the natural grain and color of the wood [6].

The adhesion of paints and adhesive to a substrate is closely related to the free surface energy, with higher surface energy promoting better adhesion [31,32]. In general, homogeneous surfaces possess higher surface energy, which can present challenges when dealing with porous oak wood [12]. For example, the adhesion of beech is higher than that of oak [33]. Coatings that possess good wetting properties tend to adhere better to rough surfaces [31]. Conversely, smooth surfaces have poor wetting properties, making it difficult to determine the most suitable surface treatment for all types of coatings [31,34]. Research has shown that achieving a high-quality and durable coating is more attainable on level and preferably homogeneous substrates. Appropriate surface preparation, such as paint penetration, leveling of surface acidity, or limiting the negative effect of vessel size, can be accomplished through the use of pore fillers [8,13,32,35].

Additionally, the effectiveness of the top layer of paint can significantly influence the durability of the modification. This is supported by studies that focus on using plasma technology to modify the substrate, testing the interaction between the modification and the coating [36]. Other studies explore the use of different coating systems to stabilize the modified substrate [37,38].

Numerous scientific studies have been conducted to investigate the impact of various coating systems on the properties of oak wood [16,19,22,32,39]. However, it is essential to note that many of these studies only describe the changes in properties before exposure, and practical aspects regarding the long-term performance of the coating system or the effects of pretreatment may be limited [34,35,36,40].

## 2. Materials and Methods

### 2.1. Wood Samples, Treatment, and Exposure

**Figure 3:**
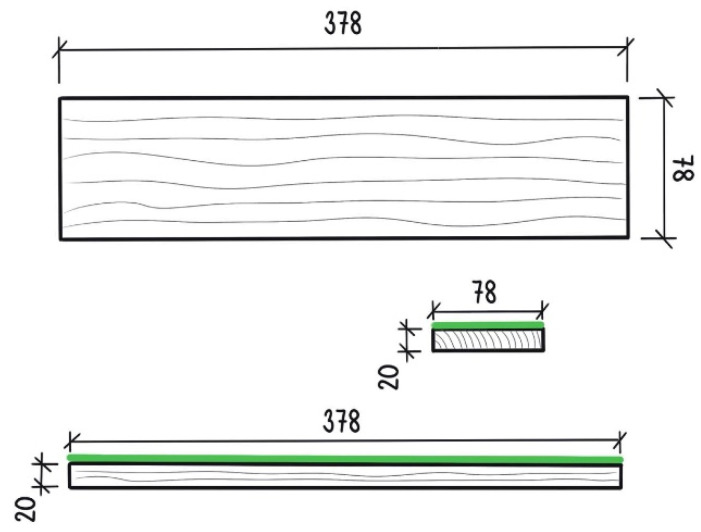
**Illustration of the sample.** The dimensions of the sample were prepared according to EN 927-3 (2020); the green line represents the coating. The dimensions are given in millimeters (mm).

Heartwood samples from English oak (*Quercus robur* L.) with dimensions 378 × 78 × 20 mm (L × T × R) (Figure 3) were prepared according to EN 927-3 (2019) from wood harvested in the Czech Republic [41,42]. Clear samples were conditioned for a relative humidity (*ϕ*) = 65 ± 5% and temperature (*t*) = 20 ± 2 °C to achieve an equilibrium moisture content (EMC) of 12%. The surfaces of the conditioned samples were sanded (120-grit) before treatment. The average density of the oak wood was 795 kg·m^−3^.

Two main sets of samples were used. The first set did not undergo pore filling treatment, and the second set underwent epoxy resin pore filling treatment. For the pore filling treatment, a colorless two-component epoxy resin (EPINAL UR 36.14), and hardener (EPINAL UH 36.14) from Acolor© (Acolor, Benesov, Czech Republic) were applied. The back and side surfaces of both sets of samples were treated with an auxiliary transparent coating, while the front surfaces were sealed with silicone glue. The surface protection was necessary to reduce wetting from sides other than those being tested. Each main set of test samples was further divided into four subsets according to the resulting surface treatment. The specific surface treatment of each individual sample is shown in Table 1. The material for the production of test samples was supplied by WoodStore©, Czech Republic, Prague.

Climatic conditions during exposure were monitored and are shown in Table 2 (http://meteostanice.agrobiologie.cz) accessed on 20 April 2023 [40].

### 2.2. Changes in Color Determination

The color parameters CIE L*a*b* [41] of the test specimens were measured after 0, 3, 6, 12, and 24 months of natural weathering (NW) using a CM-600d spectrophotometer (Konica Minolta, Osaka, Japan). The measurement was carried out at eight marked positions on each sample to ensure accurate results. Two replicates were performed for each treatment. For the observation of reflection, the specular component was included at a 10° angle and d/8 geometry, with illumination based on the D65 standard (corresponding to daylight at 6500 K). Eight measurements were carried out for each sample at each weathering time point. Color change evaluations were done in the CIE L*a*b* color space, where L* is lightness from 0 (black) to 100 (white), a* is chromaticity coordinate + 60 (red) or – 60 (green), and b* is the chromaticity coordinate + 60 (yellow) or – 60 (blue). The total color difference ΔE* [41] was subsequently calculated based on the relative changes in color (ΔL*, Δa*, and Δb*) using Equation (1). The color changes were further compared to the values described in the EN 927—3 standard (2019) [43] (Table 3).

(1) $$∆E=\sqrt{{\left(∆L^{*}\right)}^{2}+{\left(∆a^{*}\right)}^{2}+{\left(∆b^{*}\right)}^{2}}$$

### 2.3. Changes in Gloss Determination

The gloss of the different coatings was measured before and during weathering tests following ISO 2813 standard (2014) [43] using a glossmeter (MG268-F2, KSJ, Quanzhou, China). Four measurements were taken at a 60° angle on each sample after 0, 3, 6, 12, and 24 months of NW to evaluate the changes in gloss. The gloss measurements were performed in four marked areas on each sample.

### 2.4. Changes in Hydrophobicity Determination and Surface Free Energy

The contact angle of distilled water on the tangential surfaces of tested samples was measured using a goniometer (Krüss DSA 30E, Krüss, Hamburg, Germany). Ten random positions on each sample were selected to measure the contact angle. The sessile drop method was used, with 10 measurements conducted per sample before and after 3, 6, 12 and 24 months of NW. Distilled water drops with a dosing volume of 5 µL were used for the measurement, and the contact angle were determined after 5 s. The contact angle measurement helps determine the wettability of a surface. A surface is considered wettable or hydrophilic when the contact angle is between 0° and 90° and is classified as non-wettable or hydrophobic when the contact angle is between 90° and 180°.

Different standardized and non-standardized methods have been compiled for wettability measurement. One of the methods involves experimentally determining the contact angle corresponding to the steady state of the liquid on the surface of the substrate, according to Young’s Equation (2):

(2) $$\gamma_{L}*cos\theta =\gamma_{S}-\gamma_{SL}$$

γ_*S*_—surface free energy of a solid material,
γ_*L*_—surface energy of a liquid,
γ_*SL*_—surface energy at the interface of solid material and liquid,
θ—wetting angle [44].

The surface free energy was measured using the same device as the surface wettability measurement. Distilled water H_2_O and diiodomethane CH_2_I_2_ were used as the test liquids. The measurements were conducted on both the pore-filled surface and the non-filled-pore surface; cementation was measured before starting the weathering exposure. The surface free energy (SFE) calculation was calculated using the Advance program ORWK model (a model for determining SFE using the contact angle of multiple liquids, Krüss GmbH, Hamburg, Germany). The dosing volume for the sessile drop method was set at 5 μL for both measurements.

### 2.5. Visual Evaluation and Microscopic Analyses

Visual changes were monitored by scanning on a desktop scanner at a resolution of 300 DPI (Canon 2520 MFP, Canon, Tokyo, Japan) before, during and after weathering. A confocal scanning laser microscope (Lext Ols 4100, Olympus, Tokyo, Japan) was used for microscopic analysis.

### 2.6. Statistical Analyses

Statistical analyses of data were performed in MS Excel and Statistica 14 (StatSoft, Palo Alto, CA, USA) using mean values, whisker plots with mean values, and 95% two-sided confidence intervals.

## 3. Results

A visual comparison of the color change of each sample was regularly recorded and scanned. The progression of visible degradation over time is presented in Table 4. Visible color changes were noticed in all samples. The initial treatment by pore filling showed smaller color changes. However, after 24 months of aging, significant color changes were observed in all samples.

Microscopic images of the surfaces shown in Table 5 demonstrate the infestation of the wood samples by mold spores and the presence of surface fouling caused by fumes and dust particles, leading to damage to the coating system. Mold spores and surface fouling can also contribute to greying of the wood surface. The most significant difference between the filled-pore and non-filled-pore surfaces was revealed in the 12th month. The filled-pore samples showed better coating stability and better maintenance of the original color of the oak, although some initial defects were observed due to the presence of fungal spores. Molds appear as black spheres in the microscopic images presented in Table 5. This damage serves as one of the first indicators of incipient surface degradation. After 24 months of weathering exposure, the surfaces of both filled-pore, and non-filled-pore surfaces appeared relatively similar. In Table 5 are molds shown by the blue arrow and surface roughness shown by red line.

**Figure 4:**
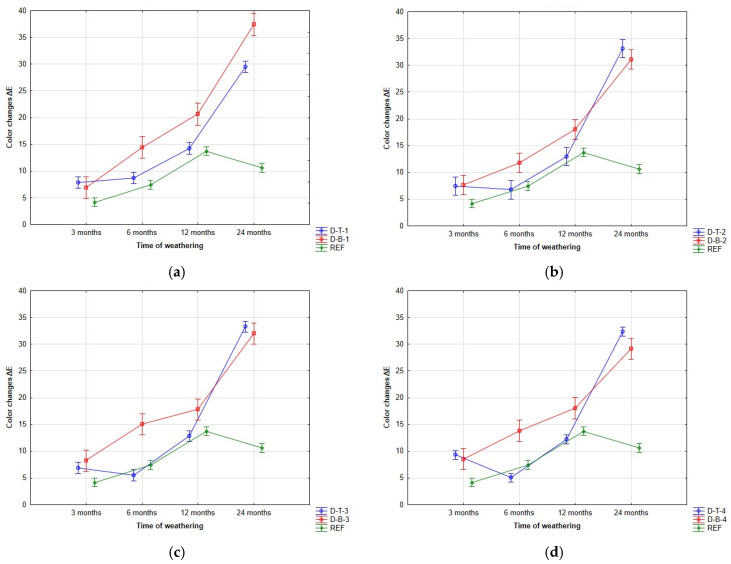
**Color changes.** This figure shows the evolution of the total color changes: (**a**) coating system 1; (**b**) coating system 2; (**c**) coating system 3; and (**d**) coating system 4.

The positive effect of pore filling in oak wood was manifested during the first 12 months of the experiment. During this period, the filled-pore samples showed minor color changes (Figure 4). The reduced color changes in the filled-pore samples were attributed to the decreased leaching of extractives, as sealing the pores limited their diffusion. The degree of color change varied depending on the type of covering paint system used, which could be attributed to differences in surface tension. Varying surface tension values can negatively affect the wetting of the surface by the paint, resulting in uneven paint layers and increased susceptibility to color changes. The positive trend of surface smoothing was observed only up to the 12th month. After 12 months of weathering exposure, noticeable degradation was observed, even in the filled-pore samples, which eventually reached comparable values after 24 months.

**Figure 5:**
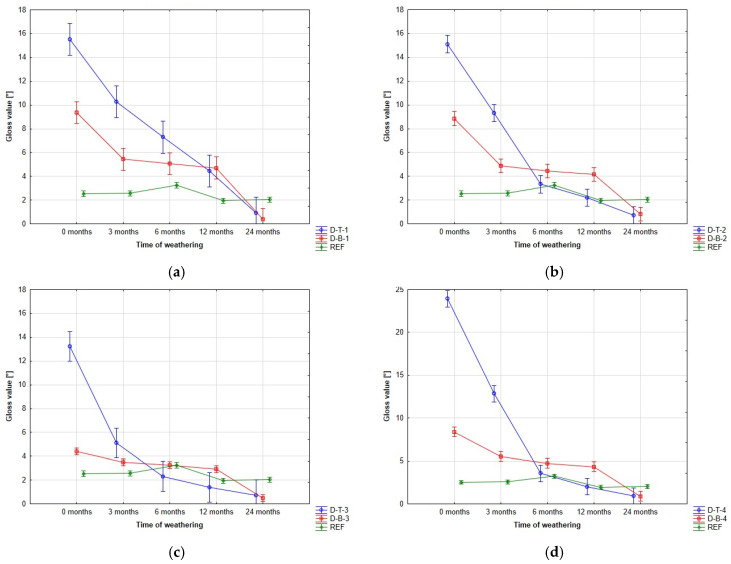
Gloss changes. This figure shows the evolution of gloss changes: (**a**) coating system 1; (**b**) coating system 2; (**c**) coating system 3; and (**d**) coating system 4.

The gloss measurement reflects the ability of the coating system to reflect light on the surface. The filled-pore samples showed higher gloss values compared to non-filled-pore samples, but there was a significant decrease in gloss values over time, resulting in similar gloss levels for both groups of samples (showed in Figure 5).

Surface energy is a factor that influences wettability, with higher values indicating better wettability, which can promote better adsorption and adhesion of the painting [7]. Table 6 shows that the filled-pore samples had a lower total surface free energy than the non-filled-pore samples. The dispersion component remained similar after surface sealing, but the polar component was reduced by half. The results indicate a reduced affinity of aqueous solutions on the modified surface [23]. The similarity in surface energy values also explains the similar wettability results observed before weathering.

**Figure 6:**
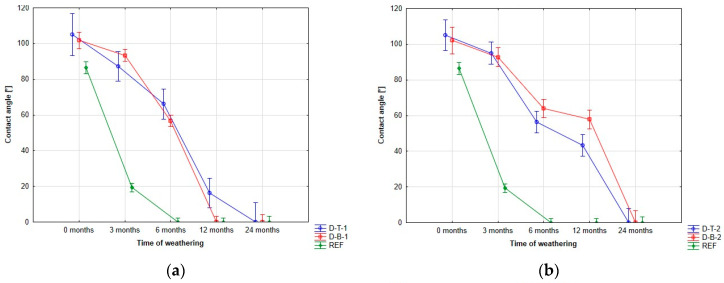

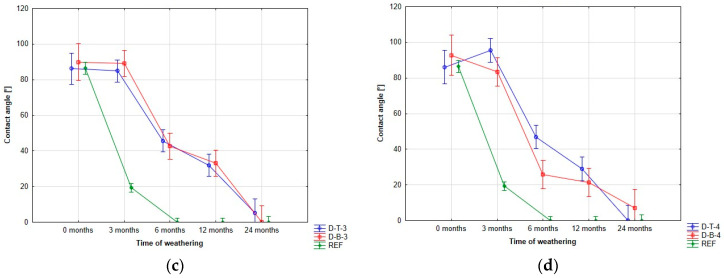
Contact angle changes. This figure shows the evolution of the contact angle: (**a**) coating system 1; (**b**) coating system 2; (**c**) coating system 3; (**d**) coating system 4.

The results show that the wettability of the surface was not significantly affected by the modification. This finding was consistent across all coating systems tested (Figure 6). There were minimal differences in surface wettability observed between the different coating systems. However, it should be noted that the contact angles of all coatings decreased after 24 months of weathering exposure, indicating a loss of hydrophobic properties and an increase in the wettability of the wood surface.

## 4. Discussion

Previous research has suggested that filling the surface pores of wood can lead to a smoother and more suitable surface for coating, resulting in more even application of paint layers [7,14,16]. The use of primer resins, such as epoxy resin, was found to be effective in filling the pores, limiting the sorption of water vapor, and preventing associated degradation effects [44,45]. Deng et al. [46] compared different types of fillers, including alkyd, one-component polyurethane, and two-component polyurethane. Alkyd filler was found to have the highest adhesive strength for oak, while one-component polyurethane filler has the lowest. Pavlič et al. [47] compared one- and two-component polyurethane and acrylic resins and concluded that the two-component polyurethane with a higher solid content had a better filling capability, similar to the results of Hiziroglu et al. [47,48].

This study found that the wetting of the surface was not noticeably affected by the sealant, except for the initial weathering exposure, in which water-based coatings showed a slightly higher contact angle than the unsealed surface, while solvent-based coatings showed the opposite trend. The lower surface energy recorded for the sealed surface suggested lower adhesion of the coating material and potentially lower durability. However, the results did not confirm lower durability, indicating that by filling large open pores in ring porous wood species, the surface energy and coating adhesion may be slightly reduced, but the local durability of the coating in area with large open pores, which tend to degrade earlier, is increased. This finding was supported by the study’s observations regarding non-sealed surfaces.

In terms of overall color change ΔE*, the sealant sets with coatings 2 and 3 had the most satisfactory color stability, and coating 4 was also at a high level. Sealing the surface had different effects on individual color coordinates. The L* coordinate tended to darken on the sealed surfaces, while it lightened on non-sealed surfaces. The a* coordinate increased with sealing, while the b* coordinate decreased. The study suggested that sealing could partially prevent the degradation of lignin and leaching of extractives. The most significant changes in individual coordinates were observed between 3 and 6 months of weathering exposure.

The application of a sealant can lead to an increase in gloss, but this increase is not permanent. The highest increase in gloss was recorded for coating system 4 using water-based coatings, reaching a gloss value three times higher than the non-sealed set. However, for the filled-pore samples, the gloss value decreased below that of the non-filled-pore sets, particularly between 3 and 6 months, and this trend continued throughout the exposure period. Visually, the filled-pore samples appeared most satisfactory, exhibiting significantly lower local color changes than the non-filled-pore samples. A visible difference between the sealed and unsealed surfaces could be observed within the first 12 months of weathering exposure.

## 5. Conclusions

Our research shows that filling the pores of wood surfaces can improve their properties by allowing a more even application of paint layers. The treatment of oak wood has a substantial impact on the service life of outdoor transparent coating systems. Oak wood can be chemically treated with preservatives to protect against mold, rot, insect attack and weathering. Pretreatment with a sealing agent has been shown to improve the durability of the coating on the wood surface. Using a base sealer such as epoxy resin can effectively fill the pores, preventing the absorption of water vapor and mitigating potential degradation effects.

The study found that the application of a sealer did not significantly impact surface wetting, except during the initial exposure to weathering. In this stage, water-based coatings showed a slightly higher contact angle on unsealed surfaces, while solvent-based coatings showed the opposite effect on sealed surfaces.

The application of a sealer had different effects on individual color coordinates. The L* coordinate tended to darken on sealed surfaces and lighten on unsealed surfaces. The a* coordinate increased with sealing, while the b* coordinate decreased. Sealing partially prevented lignin degradation and extractive leaching, and the most significant changes in individual color coordinates were observed between 3 and 6 months of exposure. Among the sealant sets, coatings 2 and 3 showed the most satisfactory colorfastness, and coating 4 also performed well.

The gloss of the coatings initially increased for the filled-pore samples, but this increase was not permanent. The highest increase in gloss was observed for coating system 4 using water-based coatings, which showed a threefold increase compared to the non-filled-pore samples. However, for the filled-pore samples, the gloss value mainly decreased between 3 and 6 months, falling below the gloss of the non-filled-pore group, and this trend continued throughout the exposure period.

Visually, the filled-pore coatings appeared more satisfactory, as they exhibited significantly lower local color changes than the non-filled-pore samples. The difference between filled-pore and non-filled-pore surfaces became evident within 12 months of weathering exposure.

## Figures and Tables

**Table 1:** Overview of sample preparation.

Wood	Code	Modification	Code	First Layer	Company	Second Layer	Company	*Coating System Number*	Number of Sample
Oak	D	Sealed pores	T	Rhenodecor Trans TIX	(DOMAŽLICE, CZECH REPUBLIC) Rhenocoll©	Protector−Plus	Adler©	**1**	**1, 2, 3**
				Rhenocryl FK 47 High Solid	Rhenocoll©	Protector–Plus	Adler©	**2**	**1, 2, 3**
				Aquawood Ligno + Base	Adler©	Aquawood Ligno + Top	Adler©	**3**	**1, 2, 3**
				Lignofix	Stachema©	Lignofix	Stachema©	**4**	**1, 2, 3**
Oak	D	Unsealed pores	B	Rhenodecor Trans TIX	Rhenocoll©	Protector–Plus	Adler©	**1**	**1, 2, 3**
				Rhenocryl FK 47 High Solid	Rhenocoll©	Protector–Plus	Adler©	**2**	**1, 2, 3**
				Aquawood Ligno + Base	Adler©	Aquawood Ligno + Top	Adler©	**3**	**1, 2, 3**
				Lignofix	Stachema©	Lignofix	Stachema©	**4**	**1, 2, 3**
Oak	D	Reference	REF			Natural wood without coating			**1, 2**

**Table 2:** Average climate values.

Year	Average Daily Temperature (°C)	Maximum Temperature (°C)	Average Relative Air Humidity (%)	Total Precipitation (mm)	Daily Incident Solar Energy (kJ/m^2^)	Maximum Solar Energy (kJ/m^2^)
2021	8.92	33.9	72.38	568.93	10,416.25	28,424
2022	10.34	37.7	68.98	472.81	11,534.65	29,648

**Table 3:** Table of color changes according to EN 927-3 standard (2020).

0.2 > Δ*E*	Invisible difference
0.2 < Δ*E* < 2	Little difference
2 < Δ*E* < 3	Color change visible with a high-quality filter
3 < Δ*E* < 6	Color change visible with a medium-quality filter
6 < Δ*E* < 12	High color changes
Δ*E* > 12	Different color

**Table 4:** Macroscopic scans of samples.

Time of Weathering (Months)					
	0	3	6	12	24
B-1	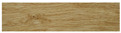	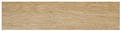	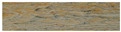	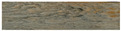	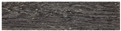
T-1	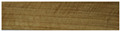	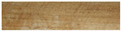	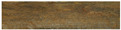	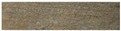	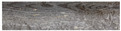
B-2	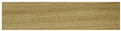	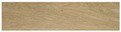	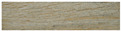	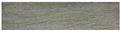	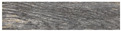
T-2	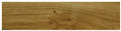	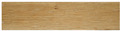	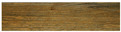	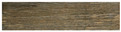	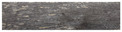
B-3	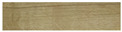	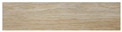	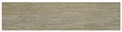	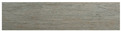	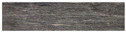
T-3	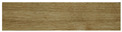	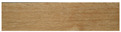	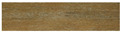	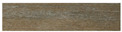	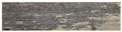
B-4	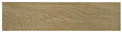	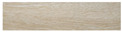	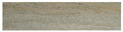	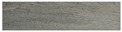	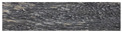
T-4	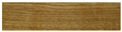	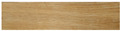	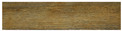	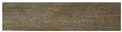	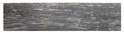

**Table 5:** Microscopic scan of sample surfaces (scale of bar is 6:100).

Natural Oak—Reference	12 Months		24 Months	
	Unmodified	Modified	Unmodified	Modified
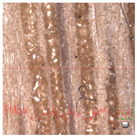	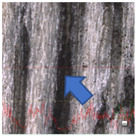	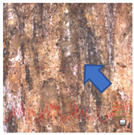	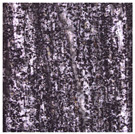	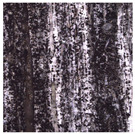

**Table 6:** Surface free energy before weathering.

	Modified Surface	Unmodified Surface
Total free surface energy (mN/m)	42.42 ± 6.85	47.12 ± 10.97
Dispersion component (mN/m)	37.48 ± 2.66	37.67 ± 2.84
Polar component (mN/m)	4.94 ± 4.19	9.45 ± 8.13

## Data Availability

Not applicable.
